# Carboxypeptidase O is a lipid droplet-associated enzyme able to cleave both acidic and polar C-terminal amino acids

**DOI:** 10.1371/journal.pone.0206824

**Published:** 2018-11-02

**Authors:** Linnea C. Burke, Hazel O. Ezeribe, Anna Y. Kwon, Donnel Dockery, Peter J. Lyons

**Affiliations:** Department of Biology, Andrews University, Berrien Springs, Michigan, United States of America; Aix Marseille University, FRANCE

## Abstract

Carboxypeptidase O (CPO) is a member of the M14 family of metallocarboxypeptidases with a preference for the cleavage of C-terminal acidic amino acids. CPO is largely expressed in the small intestine, although it has been detected in other tissues such as the brain and ovaries. CPO does not contain a prodomain, nor is it strongly regulated by pH, and hence appears to exist as a constitutively active enzyme. The goal of this study was to investigate the intracellular distribution and activity of CPO in order to predict physiological substrates and function. The distribution of CPO, when expressed in MDCK cells, was analyzed by immunofluorescence microscopy. Soon after addition of nutrient-rich media, CPO was found to associate with lipid droplets, causing an increase in lipid droplet quantity. As media became depleted, CPO moved to a broader ER distribution, no longer impacting lipid droplet numbers. Membrane cholesterol levels played a role in the distribution and *in vitro* enzymatic activity of CPO, with cholesterol enrichment leading to decreased lipid droplet association and enzymatic activity. The ability of CPO to cleave C-terminal amino acids within the early secretory pathway (*in vivo*) was examined using *Gaussia* luciferase as a substrate, C-terminally tagged with variants of an ER retention signal. While no effect of cholesterol was observed, these data show that CPO does function as an active enzyme within the ER where it removes C-terminal glutamates and aspartates, as well as a number of polar amino acids.

## Introduction

Metallocarboxypeptidases (CPs) are found in most organisms and are expressed in a wide variety of tissues [1–3]. They catalyze the removal of C-terminal amino acids from substrate peptides and proteins, many having specificity for aliphatic/aromatic or basic C-terminal amino acids (CPA-like or CPB-like enzymes, respectively) [4, 5]. Many of these CPs are placed in the MEROPS M14 family of enzymes [6] and categorized as funnelins due to sequence and structural features [4]. Of these funnelin CPs, a number are secreted from the pancreas and are involved in the digestion of dietary proteins and peptides [7]. Other CPs are involved in the maturation of neuropeptides within the secretory pathway [8–10] or in the modulation of extracellular signaling pathways [11–13]. More recently, a class of cytosolic CPs has been identified with acidic C-terminal specificity that is responsible for the modification of tubulin [14, 15]. Several members of the CP family are thought to be inactive due to the lack of a number of key catalytic residues [16].

A number of years ago a survey of the human genome resulted in the identification of another carboxypeptidase with similarity to the pancreatic/digestive CPs, carboxypeptidase O (CPO) [17]. While other digestive CPs had a prodomain thought to be necessary for folding and regulation [18, 19], CPO lacked this feature and was predicted to be an inactive carboxypeptidase homolog. It has now been shown that CPO produces a fully functional enzyme even in the absence of a prodomain, is GPI-anchored, and is expressed on the surface of intestinal enterocytes where it likely processes dietary proteins and peptides [20, 21]. The ability of CPO to cleave C-terminal acidic amino acids suggests that CPO complements the functions of CPA and CPB in the digestion of dietary proteins [20].

Although the expression of CPO is highest in the small intestine, transcripts have also been identified in brain, ovary, spleen, and lymphoid tissues [20]. In all of these tissues, CPO may function in the extracellular space; immunohistochemistry of human ileum showed CPO on the apical membrane. However, these immunohistochemical experiments also showed ample signal intracellularly, suggesting that CPO may spend a significant amount of time within cells [20]. In a more artificial system, that of stably transfected Madin-Darby canine kidney (MDCK) cells, CPO is found on both the plasma membrane and intracellularly [20]. The broad pH optimum of CPO suggests that it is not effectively regulated by pH like many other CPs [22–24] and might have a role within intracellular acidic compartments, while its lack of a prodomain suggests that CPO is not regulated through proteolysis. All of these items support the possibility that CPO has a broader function than just extracellular processing of dietary peptides. The function of CPO may include the regulation of proteins as they journey through the secretory pathway of a variety of cells and tissues.

In this study, we set out to investigate the intracellular distribution and function of CPO. Using a cell culture system, we found that CPO interacts with lipid droplets from its position on the lumenal leaflet of the ER membrane and that this interaction with lipid droplets is regulated by membrane cholesterol levels. Using an *in vivo* activity assay, we have shown that CPO is enzymatically active within the ER, and finally have used this assay to characterize the *in vivo* substrate specificity of CPO.

## Materials and methods

### Cell culture

MDCK and HEK293T cells (ATCC) were cultured in Dulbecco’s Modified Eagle’s Medium (DMEM) supplemented with 10% fetal bovine serum (FBS) and penicillin/streptomycin at 37°C and 5% CO_2_. All incubations with (2-Hydroxypropyl)-β-cyclodextrin were in serum-free DMEM, while incubations with water-soluble cholesterol were in normal growth medium. Stably-expressing MDCK cell lines were previously described [20]. MDCK cells were differentiated into polarized epithelial cells by growing at confluence for 4–6 days with media changes every two days. Differentiation was confirmed by immunocytochemistry for tubulin, showing the presence of primary cilia.

### Plasmid preparation

Plasmids were prepared using the non-ionic detergent (NID) method as described [25].

### Transfection

Cell transfection was performed using polyethylenimine (PEI). Cells were grown in 60 mm dishes to 50% confluency. Plasmid DNA (5 μg) was added to 500 μl serum-free medium, followed by the addition of 15 μl of 1 μg/μl PEI (25kD linear from Polysciences). After incubating at room temperature for 15 minutes, the mixture was added to cells.

### Immunofluorescence

MDCK cells were cultured on coverslips coated with poly-D-lysine (Sigma). Cells were washed with phosphate-buffered saline (PBS), fixed in 4% paraformaldehyde for 15 min, and then permeabilized for 15 min in 0.1% Triton X-100 in PBS. After 1 hour of blocking in 5% bovine serum albumin in PBS, cells were immunostained for 1 hour with rabbit RP3-CPO (Triple Point Biologics; 1:1500 dilution), rat anti-Nup98 (clone 2H10, Sigma, 1:1500 dilution), mouse anti-Caveolin-1 (clone CAV-1, Sigma, 1:1000 dilution), mouse anti-tubulin (clone DM1A, Cell Signaling Technology, 1:1000 dilution), mouse anti-58K Golgi protein (Abcam, 1:100 dilution), and/or mouse anti-GFP (clone 11E5, Fisher Scientific, 1:500 dilution) antibodies. The cells were washed three times with PBS and then incubated with Alexa Fluor 488 and/or Alexa Fluor 555 conjugated secondary antibodies (Cell Signaling Technology, 1:1000 dilutions) for 1 hour. After 3 washes with PBS, coverslips were inverted on a slide with 8 μl of buffered glycerol with antifade (1 mg/ml p-phenylenediamine hydrochloride, 10 mM Tris-HCl, pH 9.0, 90% glycerol). Staining for lipid droplets was performed for 30 minutes with BODIPY 493/503 (Invitrogen) diluted in PBS. Cells were then mounted in PBS containing antifade (see above) and 1 μg/ml DAPI. Imaging was performed with a Leitz Laborlux D microscope equipped with a SPOT RT cooled CCD monochrome camera.

### Western blotting

Proteins were resolved by SDS-PAGE on 10% gels and transferred to nitrocellulose. Western blotting was performed according to standard protocol with rabbit RP3-CPO (Triple Point Biologics; 1:4000 dilution) primary antibody and horseradish peroxidase-conjugated secondary antibody (Cell Signaling Technology, 1:2000 dilution). Images were obtained using LumiGLO chemiluminescent reagent (Cell Signaling Technology) and quantified using ImageJ (NIH).

### Carboxypeptidase enzyme assay

Carboxypeptidase activity was quantified by incubating 10 μl protein extracts (in 20 mM Tris pH 8.0, 150 mM NaCl, 1% Triton X-100, and 0.1 mM PMSF) with 100 μl of 0.5 mM FA-Glu-Glu chromogenic substrate (Bachem; dissolved in 50 mM Tris-HCl, 150 mM NaCl, pH 7.5) at 37°C in a 96 well plate. Absorbance at 340 nm was measured every minute for 30 minutes, and the rate of reaction determined. Activity was indicated by a decrease in absorbance. Catalytic constants were calculated using a web-based application found at ic50.tk/kmvmax.html.

### Luciferase assay

Plasmids expressing *Gaussia* luciferase, wild-type and C-terminally KDEL- or RTDL-tagged, were a generous gift from Dr. Brandon Harvey (NIH). For experiments examining the role of cholesterol in modifying CPO activity, MDCK cells were trypsinized 6 hours following transfection and transferred to 24-well plates. The next day, following treatment with water-soluble cholesterol (Sigma) or (2-hydroxypropyl)-β-cyclodextrin (HPCD; Sigma) for 5 hours in 300 μl regular growth media, media was collected and cells were harvested in 100 μl lysis buffer (50mM Tris (pH 7.5), 150mM NaCl, 1% NP40, and protease inhibitors). Experiments examining the C-terminal substrate preferences of CPO were initiated by the transfection of HEK293T cells in 24-well plates. Media (~500 μl) and lysates (100 μl) were collected following 2 days of incubation. Luciferase activity of 5 μl of each sample was measured on a TD-20/20 luminometer (Turner Designs) upon injection of 100 μl of 8 μM coelenterazine (Regis) in PBS.

### Site-directed mutagenesis

Point mutations and insertions were created using the PfuUltra II Fusion HS polymerase (Agilent Technologies) with the QuikChange site-directed mutagenesis method (Stratagene). Primer sequences available upon request. All mutations were confirmed through sequencing by GenScript (NJ, USA).

## Results

### Carboxypeptidase O associates with lipid droplets

**Fig 1:**
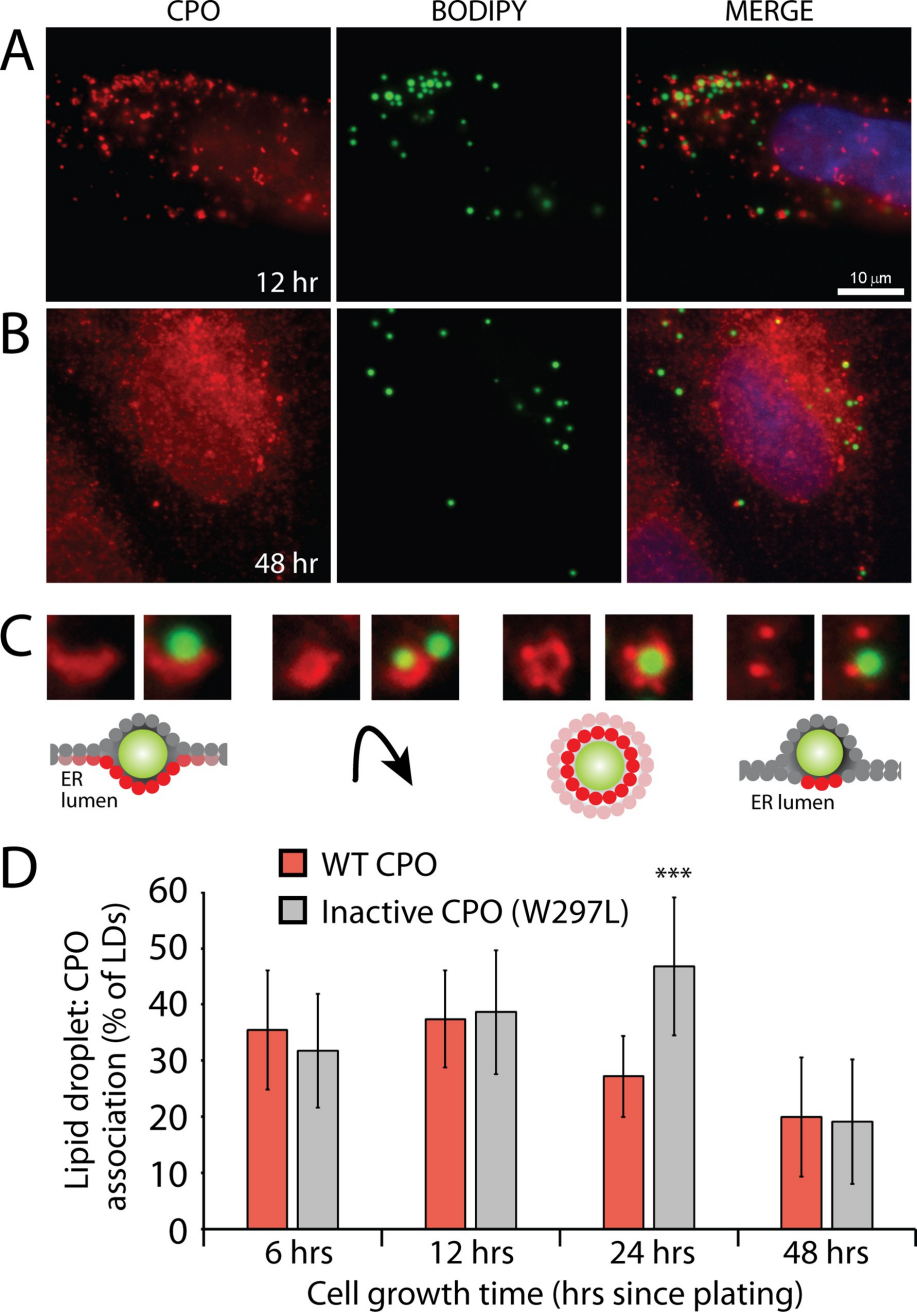
CPO associates with lipid droplets upon early exposure to nutrients. Lipid droplets and CPO expressed in stably-transfected MDCK cells were visualized by immunofluorescence microscopy. MDCK cells were fixed 12 hours (A) or 48 hours (B) following plating. (C) Lipid droplets (green) were often seen cupped by CPO (red; left), surrounded by CPO (right middle), or tightly associated with smaller CPO puncta (right). Due to the GPI-anchoring of CPO, CPO is present on the lumenal leaflet of the ER membrane. The likely position of CPO relative to a lipid droplet is illustrated. (D) The mean percentage of lipid droplets that associated closely with CPO puncta was quantified. Each sample included at least 10 fields of view from two separate experiments. *** *p* < 0.001, by student’s *t* test.

The subcellular distribution of CPO was examined by immunofluorescence microscopy of stably-transfected MDCK cells. CPO was distributed in a punctate pattern throughout the cytoplasm (Fig 1A) when cells were fixed within 6–24 hours from plating. Longer periods of incubation (48 hours; Fig 1B) resulted in a broader ER localization, consistent with the presence of an N-terminal ER signal peptide. This change in distribution appeared to be dependent on the time since addition of nutrient-rich media, as cells for which the media was changed within the previous 24 hours presented the punctate pattern of CPO expression regardless of the time of prior growth (results not shown). CPO puncta were commonly seen cupping lipid droplets, sometimes encircling lipid droplets, and often as smaller points in close association with lipid droplets (Fig 1C). Not all lipid droplets were found associated with CPO puncta; 35–37% of lipid droplets were associated with wild-type CPO at 6- and 12-hour time points, dropping gradually to 20% at 48 hours (Fig 1D). Analysis of the association of lipid droplets with an inactive CPO mutant (W297L; see S1 Fig) showed an increase from 32% at 6 hours to 47% at 24 hours, followed by a drop to 20% as seen for wild-type CPO (Fig 1D). The level of association of lipid droplets with inactive W297L CPO at 24 hours showed a statistically significant difference from that of wild-type CPO.

**Fig 2:**
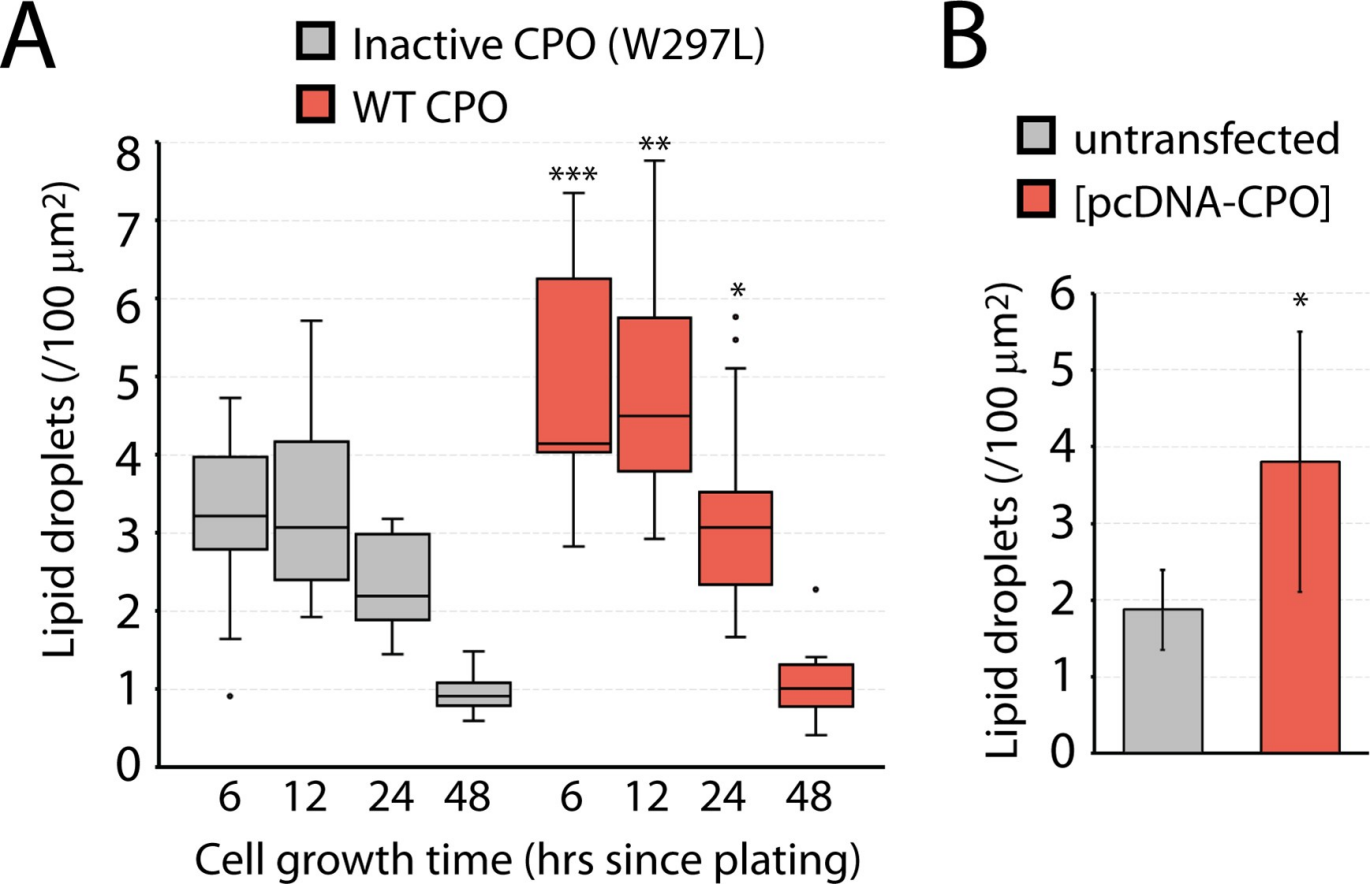
Lipid droplet quantity is increased upon CPO expression. A) The number of lipid droplets per 100 μm^2^ of cell area was counted in stably-transfected MDCK cells. Each sample included at least 10 fields of view from two separate experiments and is presented as the median value in a box and whisker plot. Cell area was determined using ImageJ. B) Transiently-transfected MDCK cells were compared with untransfected neighboring cells at 12 hours after transfection. * *p* < 0.05; ** *p* < 0.01; *** *p* < 0.001, by student’s *t* test in comparisons of cells expressing active CPO and control.

The observed pattern of CPO distribution was consistent with the accumulation of CPO on the lumenal leaflet of the ER membrane during the development of a lipid droplet in the intermembrane space (see Fig 1C). To test the possibility that CPO was involved in lipid droplet formation, the number of lipid droplets present per cell area were counted in cells stably expressing the inactive W297L CPO mutant, and in those expressing wild-type CPO. Median numbers of lipid droplets in cells expressing the inactive CPO mutant at 6 and 12 hours were approximately 3 lipid droplets per 100 μm^2^ cell area (Fig 2A). In cells expressing wild-type CPO this increased to 4–5 lipid droplets per 100 μm^2^ cell area. At later time points, lipid droplet numbers in cells expressing wild-type or inactive CPO rapidly dropped off to 1 per 100 μm^2^ cell area. A similar analysis of lipid droplet quantity was done with transiently transfected MDCK cells, to control for any selection that may occur during the process of isolating stably-expressing cell lines. In this case, the control cells were untransfected cells surrounding the less abundant transiently transfected cells. A two-fold increase in the number of lipid droplets was observed 12 hours following transient transfection of CPO (Fig 2B).

**Fig 3:**
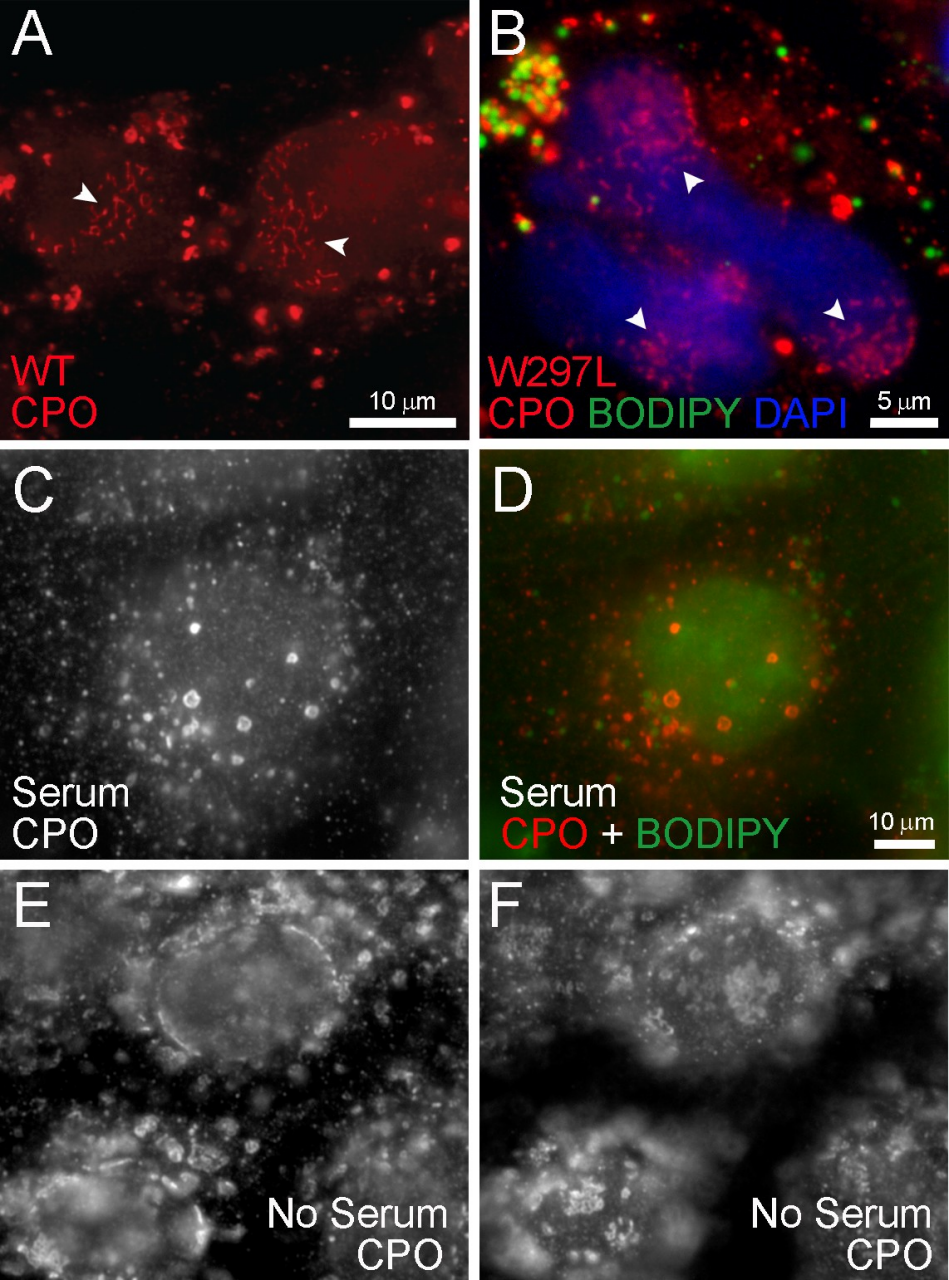
CPO is often found on the nuclear envelope and aggregates upon serum starvation. MDCK cells stably expressing CPO were fixed and immunostained with an antibody to CPO (red in A, B and D; white in C, E,F) and with BODIPY 493/503 to detect lipid droplets (green in B and D). Both wild-type CPO (A) and the inactive W297L CPO mutant (B) were often detected in a reticular pattern on the nuclear envelope. This nuclear envelope association was dramatically increased upon serum starvation. Control cells presented the usual punctate pattern with some lipid droplet association (C, D), while serum starvation for 24 hours resulted in large aggregates of CPO on the nuclear envelope (see nuclear cross-section in E and nuclear surface in F).

In a portion of cells stably expressing CPO, both wild-type and inactive W297L, CPO was expressed in a wavy or reticular pattern on the nuclear envelope (Fig 3A and 3B). This association of CPO with the nuclear envelope was observed by its conformation to the surface of the nucleus as seen by autofluorescence (Fig 3A), DAPI staining (Fig 3B), and co-staining with an antibody to the nuclear pore protein, Nup98 (not shown). This pool of nuclear envelope-associated CPO was not typically associated with visible lipid droplets (see Fig 3B). When cells were serum-starved, CPO was observed in dense clusters and whorls often on the surface of the nucleus, consistent with ER structures previously described as organized smooth ER (OSER; Fig 3C–3F) [26, 27]. No lipid droplets were observed upon serum starvation.

### CPO lipid droplet association is regulated by membrane cholesterol levels

GPI-anchored proteins are often found within lipid rafts or detergent-resistant membrane domains [28]. Immunocytochemistry was performed to determine if the observed distribution of CPO in stably transfected MDCK cells was similar to that of known lipid raft proteins. Caveolin-1, a well-characterized component of specialized lipid raft-like structures termed caveolae [28], was detected only weakly by immunocytochemistry, mostly on the cell surface and in a perinuclear domain suggestive of the Golgi apparatus, and not colocalized with CPO (not shown). The Golgi apparatus is known to be rich in cholesterol [29]; however, CPO did not co-localize with a marker of the Golgi apparatus (S2 Fig). Erlin-1 and -2 are proteins found to localize to ER lipid rafts [30]. Transient transfection of a plasmid expressing Erlin-2-GFP into CPO-expressing MDCK cells resulted in some co-localization with CPO, particularly on the nuclear envelope (S2 Fig). Transiently transfected Erlin-2-GFP, however, had a more ER-like distribution. It could be that the expression levels of Erlin2-GFP in this experiment were too high to detect a lipid raft distribution.

**Fig 4:**
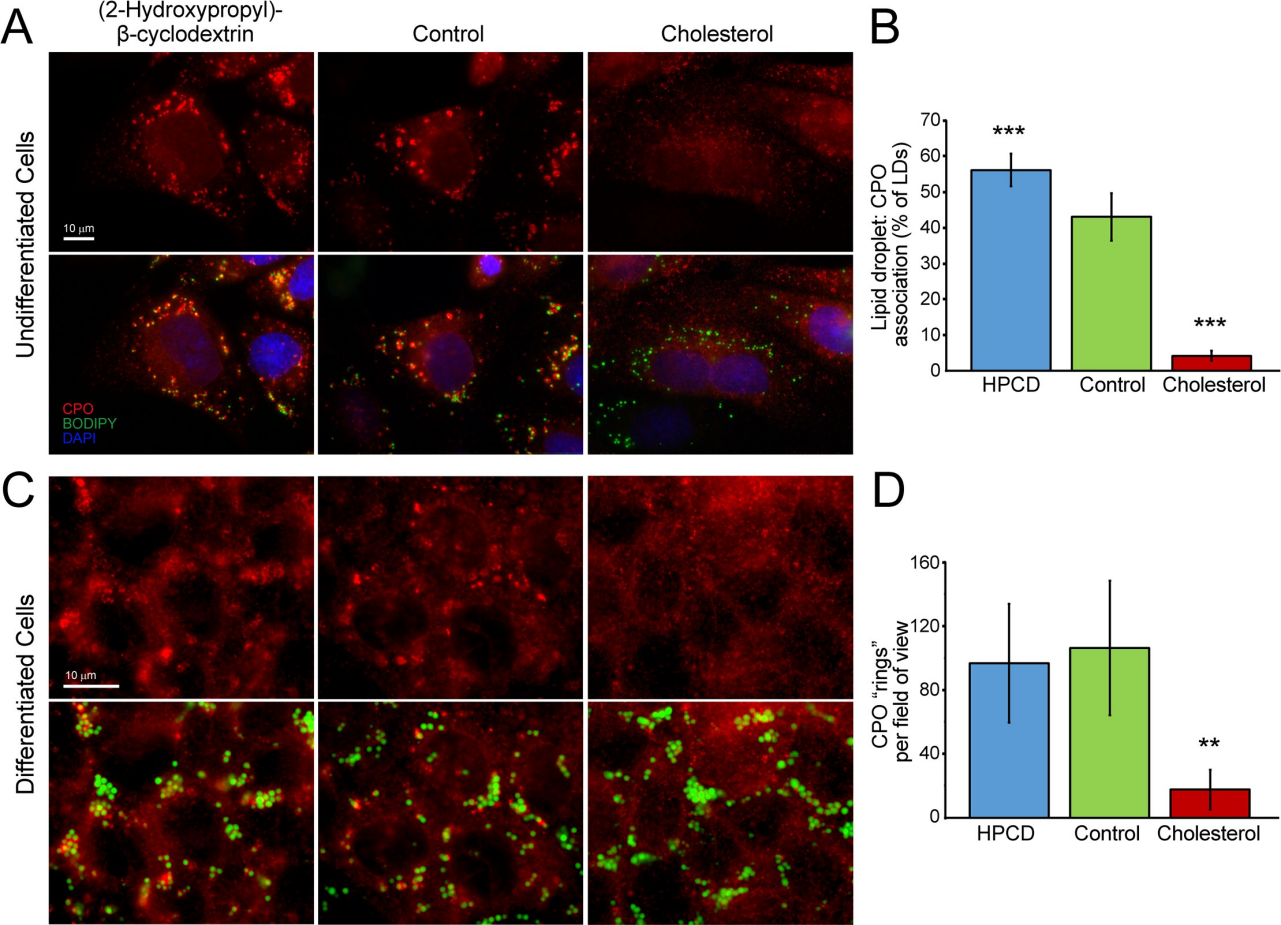
The association of CPO with lipid droplets is regulated by membrane cholesterol content. (A) MDCK cells, stably expressing CPO, were incubated for 1 hour with serum-free medium containing 5% (2-Hydroxypropyl)-β-cyclodextrin, normal growth medium (control), or growth medium containing 0.6 mg/ml water soluble cholesterol. Lipid droplets were labeled with BODIPY and observed together with CPO by immunofluorescence microscopy. (B) The mean percentage of lipid droplets that associated closely with CPO puncta was quantified. n = 9. *** *p* < 0.001 (C) MDCK cells were first differentiated to an epithelial morphology. (D) The average number of CPO “rings” observed per field of view. n = 7. ** *p* < 0.01.

There have been some reports of caveolins targeted to lipid droplets [31, 32], suggesting that lipid droplets may be surrounded by a cholesterol-rich membrane. Although we were not able to clearly show co-localization of CPO with caveolin-1 on lipid droplets, one would expect that the distribution of a protein found in such a membrane might change upon enrichment or depletion of cellular cholesterol. Stably transfected MDCK cells were treated for one hour with (2-hydroxypropyl)-β-cyclodextrin (HPCD) in serum-free media to deplete cholesterol, or with water soluble cholesterol in normal growth media to enrich membrane cholesterol, and the distribution of CPO was analyzed by immunocytochemistry. Cholesterol depletion resulted in aggregation of CPO around 56% of lipid droplets. This association decreased to 43% of lipid droplets in control cells, while cholesterol enrichment showed a broad distribution of CPO with only 4% of lipid droplets clearly associated with CPO (Fig 4A and 4B), possibly due to the broader availability of cholesterol-rich membranes to interact with. Similar experiments were performed with stably transfected MDCK cells that were differentiated into an epithelial morphology. In all samples, rings of CPO were detected that nearly always surrounded lipid droplets. However, there were many fewer of these CPO “rings” in cells treated with cholesterol than in control or cholesterol-depleted cells (Fig 4C and 4D).

### Cholesterol modulates enzymatic activity of CPO *in vitro* but not *in vivo*

**Fig 5:**
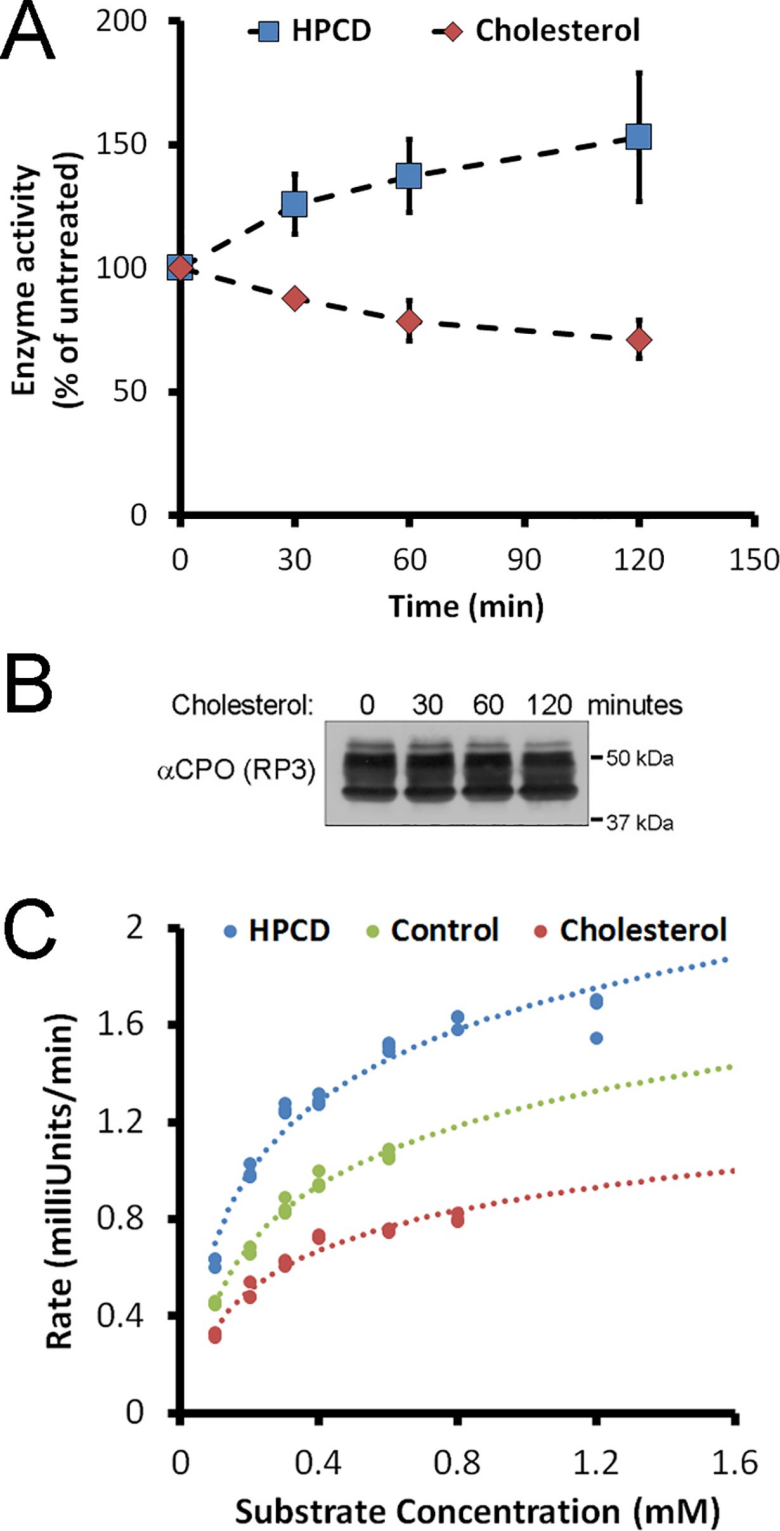
Membrane cholesterol levels modulate enzymatic activity of CPO *in vitro*. (A) MDCK cells stably expressing CPO were treated for the indicated times with cholesterol or (2-Hydroxypropyl)-β-cyclodextrin (HPCD). Lysates were incubated with 0.5 mM FA-EE at pH 7.5 and 37°C and initial rates of reaction were determined and plotted relative to a no treatment control. n = 3 (B) Lysates from cells treated with cholesterol were analyzed for CPO quantity by western blotting. No change in CPO quantity was observed. (C) Lysates of cells treated for 2 hours with cholesterol or HPCD were incubated with a range of FA-EE substrate concentrations at pH 7.5 and 37°C. Michaelis-Menton plots indicated that the change in activity was due to a change in V_max_, but not in K_M_.

Our observation that the distribution of CPO changed with cholesterol levels, likely through an association of CPO with cholesterol-rich membranes, prompted us to ask if enzymatic activity of CPO was also changed upon this association, as has been observed for other GPI-anchored proteins [33]. This could be important in regulating the activity of CPO at the lipid droplet and/or within the ER. MDCK cells expressing CPO were treated with HPCD or cholesterol for 0 to 120 minutes. Lysates were collected and incubated with 0.5 mM FA-EE to determine initial rate of reaction. Increased CPO enzymatic activity was observed in cell lysates following the incubation of cells with HPCD for increasing amounts of time (Fig 5A, blue). In contrast, decreased enzymatic activity was observed in lysates following the incubation of cells with cholesterol for increasing amounts of time (Fig 5A, red). This decrease in enzymatic activity with cholesterol was not caused by decreased amounts of CPO due to cell death or decreased expression, as shown by western blotting (Fig 5B). Michaelis-Menton analysis showed no change in the K_M_ of CPO upon treatment of cells with either HPCD or cholesterol for 2 hours (Fig 5C); K_M_ values averaged 210 ± 26 μM for all. V_max_, however, changed from 1.0 mU/min for CPO from cholesterol-treated cells to 1.5 mU/min for CPO from untreated control cells and 2.0 mU/min for HPCD-treated cells.

**Fig 6:**
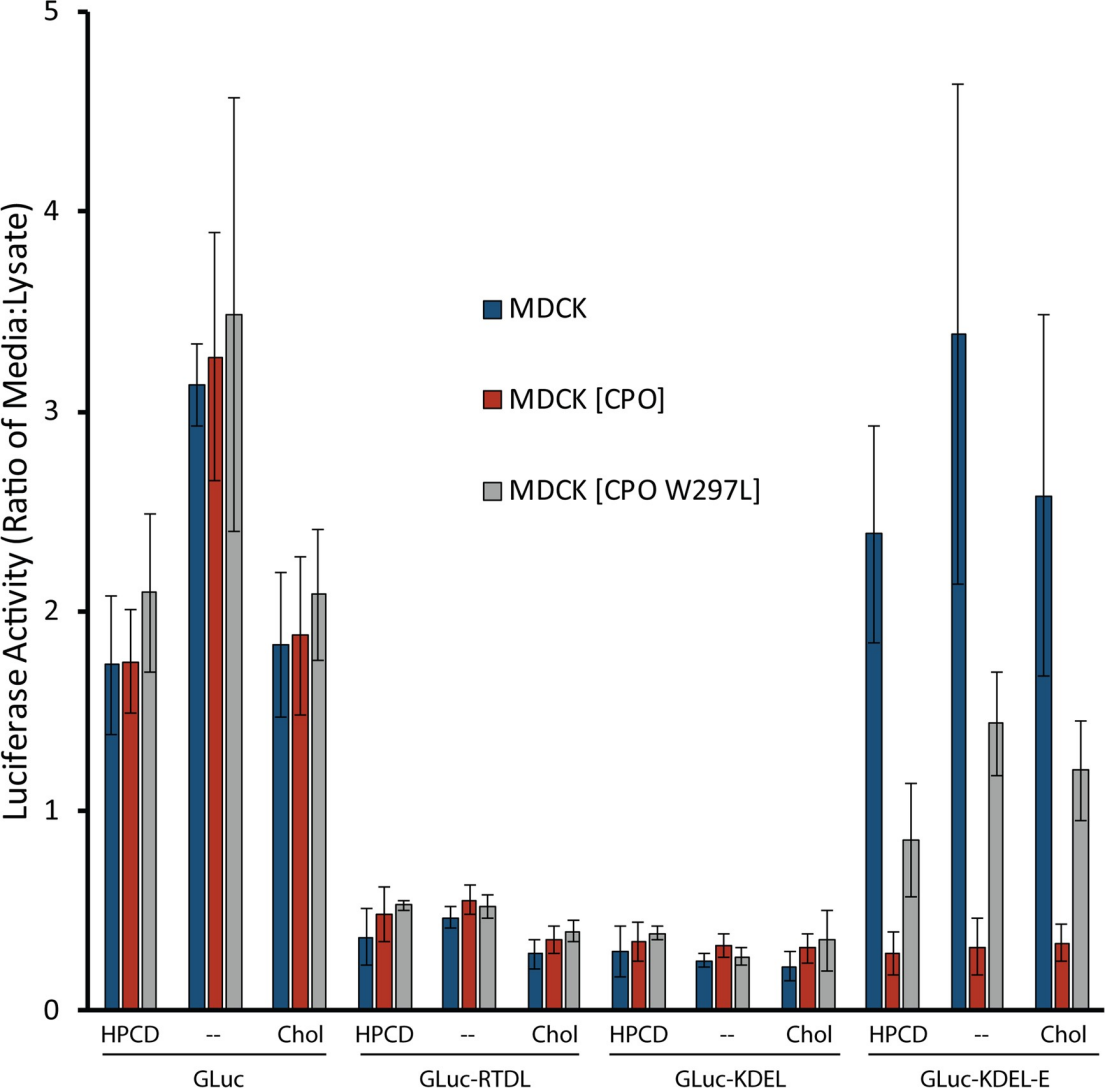
CPO is an active enzyme within the early secretory pathway, as determined by ER retention of a KDEL-E-tagged *Gaussia* luciferase. Control (stably transfected with empty vector) MDCK cells or those stably expressing active (wild-type) or inactive (W297L) CPO were transiently transfected with plasmids expressing the indicated *Gaussia* luciferase variants. Six hours following transfection, cells were trypsinized and transferred to 24-well plates. The next day cells were incubated for 5 hours with cholesterol or HPCD. Luciferase activities of media and cell extracts were measured. The ratio of luciferase activity in media to that in the lysate is shown. Error bars indicate standard deviation. n = 6–12.

This result suggested an effect of cholesterol on the activity of CPO or its accessibility to substrates, and so we wished to determine if this might be relevant *in vivo*. We needed an *in vivo* activity assay that would enable us to observe the activity of CPO within undisturbed cells. Plasmids were obtained that encoded the naturally secreted *Gaussia* luciferase (GLuc) or variants containing C-terminal ER retention signals (GLuc-KDEL and GLuc-RTDL)[34]; the C-terminal cleavage of these signals by a carboxypeptidase such as CPO would render them ineffective as retention signals and allow the luciferase protein to be secreted. These plasmids were transiently transfected into control MDCK cells or those stably expressing either wild-type CPO or an inactive variant of CPO, W297L (see S1 Fig), and then treated with cholesterol or HPCD for 5 hours prior to collection of media and lysis of cells. As previously reported [34], GLuc was predominantly secreted while the C-terminally KDEL- and RTDL-modified versions of GLuc were retained intracellularly (Fig 6). No significant change in secretion was observed when these reporters were expressed in cells also expressing wild-type CPO, or the inactive W297L CPO mutant, suggesting that CPO did not cleave the C-terminal leucine of these ER retention signals. Previous *in vitro* experiments support the inability of CPO to cleave C-terminal hydrophobic amino acids such as leucine [20]. In addition, no role for cholesterol in the regulation of CPO activity or specificity was detected—a slight decrease in secretion was observed when cells were both depleted and enriched for cholesterol.

Another approach was taken to develop an *in vivo* activity assay for CPO. Site-directed mutagenesis was used to insert one glutamate, the preferred substrate of CPO [20], C-terminal to the KDEL ER retention signal already present on the C-terminus of GLuc. This newly added C-terminal amino acid effectively inactivated the retention signal, resulting in the secretion of this modified GLuc (GLuc-KDEL-E) when transiently expressed in control MDCK cells (Fig 6). However, when transfected into MDCK cells stably expressing wild-type CPO, GLuc-KDEL-E was largely retained within the cell (Fig 6), suggesting that cleavage of the C-terminal glutamate led to re-activation of the KDEL ER retention signal. The inactive CPO mutant, W297L (see S1 Fig), was used as an additional negative control. Upon transfection of GLuc-KDEL-E into cells stably expressing this inactive mutant, a level of secretion was observed that was greater than that seen in cells not expressing CPO, but less than observed from cells expressing wild-type CPO, suggesting that CPO may have some non-enzymatic effect on secretion in this system. As this difference was not seen in GLuc- and GLuc-KDEL-transfected cells, it appears to be specific to the GLuc-KDEL-E substrate of CPO. No difference in secretion of GLuc-KDEL-E was observed when any of these cells were treated with cholesterol or HPCD to deplete cholesterol, suggesting that changes in cholesterol levels do not alter the enzymatic activity of CPO *in vivo*, in contrast to that suggested *in vitro* (see Fig 5).

### CPO cleaves C-terminal acidic and polar amino acids within the early secretory pathway

**Fig 7:**
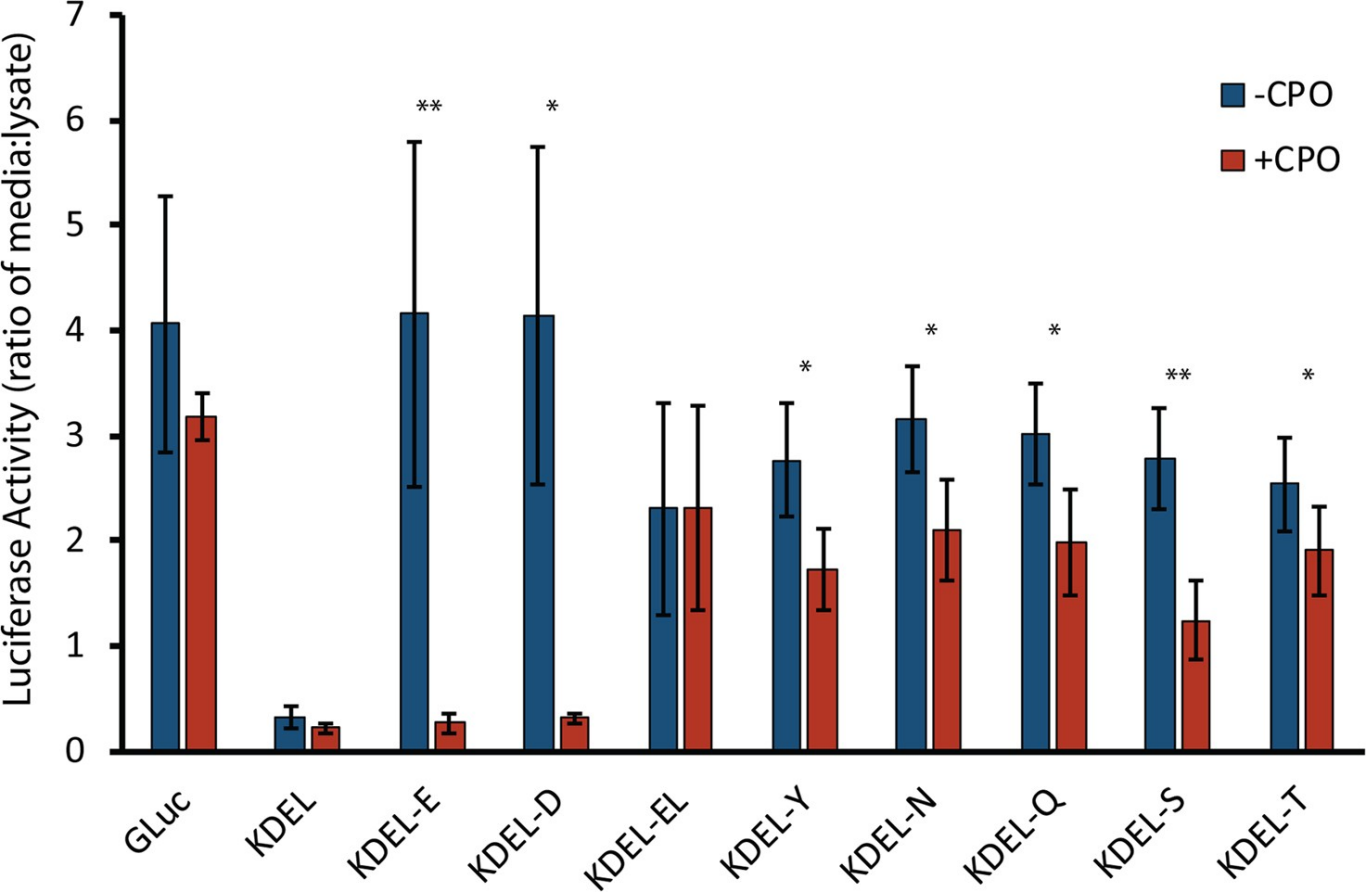
CPO cleaves both acidic and polar amino acids within the early secretory pathway. HEK293T cells were transfected with empty vector (-CPO) or pcDNA-CPO (+CPO), along with plasmids expressing secreted *Gaussia* luciferase (GLuc), intracellularly retained GLuc-KDEL, or indicated C-terminal variants. Luciferase activities of media and cell extracts were measured. The ratio of luciferase activity in media to that in the lysate is shown. Error bars indicate standard deviation. * p < 0.05; ** p < 0.01, as determined by the Mann-Whitney U Test. n = 4–7.

Most notably, the above data confirms that CPO is functional as an enzyme within the early secretory pathway, and suggests a method to examine the intracellular substrate specificity of CPO in living cells. Previous *in vitro* analyses of CPO substrate specificity indicated that CPO is able to cleave C-terminal glutamate [20]; cleavage of aspartate was recently demonstrated *in vitro* [21]. CPO is unable to cleave C-terminal basic or hydrophobic residues, with the exception of a very weak ability to cleave C-terminal alanine, and the cleavage of polar amino acids has not yet been investigated [20]. In order to investigate the *in vivo* substrate specificity of CPO, GLuc-KDEL was C-terminally modified by the addition of the single amino acids aspartate, tyrosine, asparagine, glutamine, serine, threonine, and the glutamyl-leucine dipeptide. Plasmids encoding these proteins were transiently transfected into HEK293T cells, along with empty vector or plasmids expressing CPO. A large decrease in the secretion of GLuc-KDEL-E was observed upon co-expression of CPO (Fig 7), similar to that seen in MDCK cells (see Fig 6). Similar results were obtained for GLuc-KDEL-D (Fig 7). No change was observed in the secretion of GLuc-KDEL-EL upon co-transfection with CPO, consistent with the inability of CPO to cleave leucine. Each of the GLuc-KDEL constructs modified with polar C-terminal residues showed a significant decrease in secretion upon co-transfection with CPO, with serine being the better substrate, threonine being the worst, and tyrosine, asparagine, and glutamine falling in between (Fig 7).

Our data suggest that CPO is able to cleave substrates with acidic or polar C-terminal amino acids within the endoplasmic reticulum. Due to GPI anchoring of CPO within the ER, substrates are likely to be either type II transmembrane proteins, with C-termini within the ER lumen, or entirely luminal ER proteins (although many of these would have a C-terminal KDEL sequence and hence unlikely to be cleaved by CPO). It is unlikely that proteins associated with mature lipid-droplets would be CPO substrates, as CPO is associated with the luminal leaflet of the ER membrane, while lipid droplets are surrounded by the outer leaflet of the ER membrane. This does not exclude CPO from a role in the formation of lipid droplets. A search of UniProtKB for all human proteins and major variants that are predicted to be type II transmembrane proteins resulted in a list of 840 entries, from which 80 had C-terminal glutamate or aspartate. Of these proteins, a number have been identified in other studies as being present within lipid rafts [35], and hence are candidates for cleavage by CPO (Table 1). Our experiments with GLuc suggest that CPO is likely to cleave a variety of luminal ER proteins as well.

**Table 1:** Candidate CPO substrates.

Gene	Protein	Tissue expression#	Subcellular localization*	C-terminal amino acids
DPP6	Dipeptidyl Peptidase Like 6	Brain	Type II transmembrane, lipid rafts	KEDEEED
HS3ST2	Heparan Sulfate-Glucosamine 3-Sulfotransferase 2	Brain	Type II transmembrane, lipid rafts	FRWE
KTN1	Kinectin 1	All	Type II transmembrane, lipid rafts	QVLE
LPCAT1	Lysophosphatidylcholine Acyltransferase 1	All	Type II transmembrane, lipid rafts	KKLD
RDH11	Retinol Dehydrogenase 11	All, Prostate	Type II transmembrane, lipid rafts	LPID
SEC11A	SEC11 Homolog A	All	Type II transmembrane, lipid rafts	VHRE
XXYLT1	Xyloside Xylosyltransferase 1	All	Type II transmembrane, lipid rafts	IPED
P4HA1	Prolyl 4-Hydroxylase Subunit Alpha 1	All	ER lumen, lipid rafts	SELE
P4HA2	Prolyl 4-Hydroxylase Subunit Alpha 2	All	ER lumen, lipid rafts	TEVD
PPIB	Peptidylprolyl Isomerase B	All	ER lumen, lipid rafts	IAKE
TOR1A	TorsinA	All	ER lumen, lipid rafts	YYDD

* Predicted by UniProtKB and from RaftProt:Mammalian Lipid raft Proteome Database (lipid-raft-database.di.uq.edu.au)

# From http://www.proteinatlas.org

## Discussion

In this study we have shown that, in a stably-expressing MDCK cell model, CPO associates with lipid droplets. Due to its GPI modification, which anchors CPO to the inner leaflet of the ER membrane, CPO is not likely associated with free, cytosolic lipid droplets, as these are surrounded by a membrane monolayer derived from the outer leaflet of the ER membrane. Rather, CPO may be involved in the events leading up to the budding and formation of lipid droplets. Our data showing an increased number of lipid droplets upon CPO overexpression support this hypothesis. At least one other GPI-anchored protein, invadolysin, has been found associated with lipid droplets. Invadolysin mutation in *Drosophila* has been shown to decrease triglyceride storage and fat body development [36, 37]. In small intestinal enterocytes, in which CPO is normally expressed, CPO might be involved in the formation and/or function of chylomicrons, which bud into the lumen of the ER and thus are coated with a membrane from the inner ER leaflet. Future work will investigate this possibility.

We present evidence that CPO is associated with cholesterol-rich membranes surrounding lipid droplets. While much of the literature focuses on cholesterol-rich cell surface lipid rafts, increasing evidence has pointed toward an important role for intracellular lipid raft-like domains. Erlin-1 and Erlin-2 were first identified as components of ER lipid rafts [30] and have been more recently found to play a role in the degradation of IP3 receptors by the ER-associated degradation (ERAD) pathway [38]. Recent studies have begun to elucidate the role of lipid raft-like mitochondria-associated ER membranes (MAM) in many aspects of cell biology and in the etiology of neurodegeneration [39, 40]. We show that the distribution of CPO is affected by enriching or depleting membranes of cholesterol, as would be expected for a protein associated with cholesterol-rich membranes. This distribution is consistent with the presence of many, if not all, GPI-anchored proteins in detergent insoluble membrane domains or lipid rafts [28]. In addition to being nucleation sites for cell signaling events [28], membrane microdomains may also be either a cause of membrane curvature or recruited to sites of curvature as a consequence of that curvature [41–43]. Thus we see that cholesterol-rich membranes may both recruit CPO to forming lipid droplets and enable the membrane curvature necessary for this droplet budding.

The activity of CPO, expressed in MDCK cells and following cell lysis, was found to vary with treatments to modify membrane cholesterol levels. The inverse relationship observed between the enzymatic activity of CPO and membrane cholesterol levels could suggest that CPO undergoes a change in tertiary or quaternary structure upon association with lipid raft-like domains. However, as no cholesterol-dependent change in activity was observed *in vivo*, we believe the observed change in enzyme activity is more likely due to the effect of membrane cholesterol on the dispersion of intracellular membranes into Triton X-100-containing vesicles upon cell lysis. It has been shown that cholesterol can impact the curvature of membranes and hence vesicular fusion events [44, 45]. Lange *et al*. showed that increasing the cholesterol content of erythrocyte ghosts from 0.9 to 1.0 (ratio of moles cholesterol to moles phospholipid) resulted in a dramatic change in the sidedness of vesicles formed upon homogenization, from predominantly inside-out to entirely right-side-out [46]. The observed increase in CPO enzyme activity upon cholesterol depletion is most likely evidence of an increased formation of inside-out vesicles from ER membranes, leading to the increased exposure of CPO to substrate, hence an increased V_max_ but no change in K_M_. Regardless of the specific cause, this result is strong support for the effectiveness of our treatments in modulating membrane composition.

Whether this modulation of membrane curvature occurs *in vivo* and has any impact on the biological function of CPO is an open question. The presence of CPO at sites of lipid droplet nucleation might suggest that both the membrane curvature and presence of CPO at these locations is facilitated by an increased cholesterol content. Similar observations of enriched cholesterol in lipid droplet and associated membranes have resulted in the suggestion of a new type of membrane domain [31]. These cholesterol-rich membranes have also been observed in hepatocytes during the formation of very-low-density lipoproteins; the process is accompanied by the formation of cholesterol-rich “ApoB-crescents” partially surrounding the forming lipid droplet [47].

We were interested to know if CPO exhibited enzyme activity within these intracellular compartments. We report here the development of a simple *in vivo* activity assay for CPO, capitalizing on the ability of the KDEL C-terminal sequence to specify ER retention while a one-amino acid extension of this sequence does not. No effect of cholesterol on CPO enzyme activity was observed, at least as directed toward this artificial luminal substrate. However, this assay does show that CPO is fully active in the early secretory pathway, and considering the observed distribution of CPO and the necessity for Golgi-localized KDEL receptors to retrieve the C-terminally processed KDEL-tagged protein product, it is likely that CPO is active in the ER. To our knowledge, CPO is the only CP with activity in the ER. Some members of the metallocarboxypeptidase family lack a prodomain and exist within the secretory pathway (CPE, CPD); however, these enzymes have been shown to function in the *trans*-Golgi or secretory vesicles [8, 10]. All other members of the CPA/B subfamily of metallocarboxypeptidases contain N-terminal prodomains that are not cleaved until the *trans*-Golgi or after secretion [22, 48].

If CPO is fully functional within the ER, then many proteins that pass through the secretory pathway might be substrates, those with acidic C-termini such as listed in Table 1, as well as those with polar C-termini. Our data suggest that CPO, at expression levels present in transfected HEK293T cells, is able to cleave GLuc-KDEL-E at rates equal or greater to GLuc-KDEL-E production in these cells. This same CPO is able to cleave polar amino acids from GLuc-KDEL-X (where X is a polar amino acid), although at rates that do not reach production rates for this substrate. Therefore, the impact of CPO on any *in vivo* substrate will depend on the rate of translation of that substrate and the local concentrations of enzyme and substrate. The colocalization of enzyme and substrate on specific membranes is likely an important aspect of CPO regulation. Future work will pursue both a targeted analysis of candidate substrates as well as unbiased proteomic screens within physiologically relevant enterocyte cell systems.

## Supporting information

S1 Fig The W297L CPO mutant exhibits no enzymatic activity, although fully expressed. HEK293T cells were transfected with plasmids expressing wild-type (WT) CPO or several CPO mutants. These mutants were identified through the Catalog of Somatic Mutations in Cancer (COSMIC). (A) Following transfection, lysates were probed for CPO expression with a CPO-specific antibody by western blotting. Equal loading was confirmed by Ponceau S staining of the nitrocellulose membrane. (B) Equal amounts of lysate were also incubated with 0.5 mM FA-EE for 30 minutes at 37°C to determine enzymatic activity of each mutant, determined by the decrease in absorbance of the substrate at 340 nm upon cleavage. n = 3, error bars indicate standard error.
(TIF)

S2 Fig CPO does not clearly associate with the Golgi apparatus or ER lipid rafts. MDCK cells stably expressing CPO were fixed and immunostained with an antibody to CPO (left panels; red) and with 58K Golgi protein (A, green), and erlin-2-GFP (B, green).
(TIF)

S1 Table Raw data. (XLSX)
