# Supplementary figures and images for: Carboxypeptidase O is a lipid droplet-associated enzyme able to cleave both acidic and polar C-terminal amino acids
Source: PLoS One. 2018 Nov 2;13(11):e0206824. doi: 10.1371/journal.pone.0206824 (PMC6214572; doi:10.1371/journal.pone.0206824)

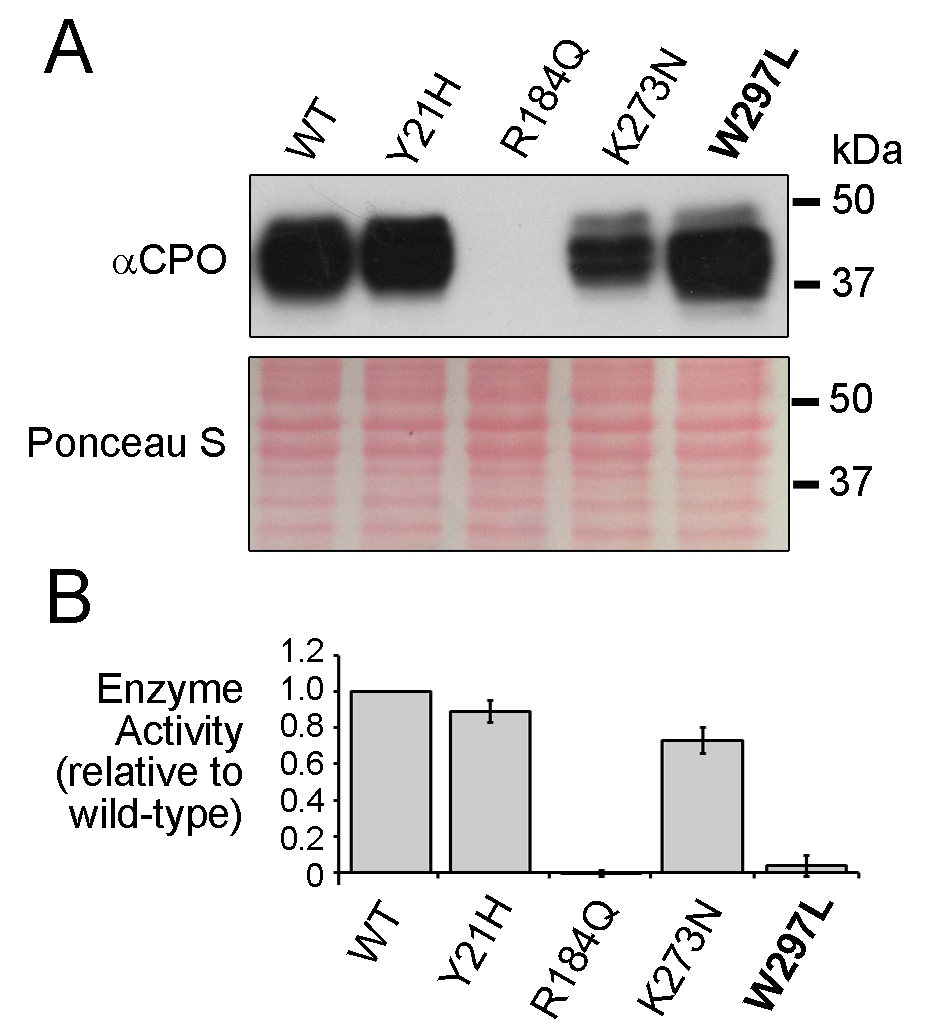

Supplement: S1 Fig — HEK293T cells were transfected with plasmids expressing wild-type (WT) CPO or several CPO mutants. These mutants were identified through the Catalog of Somatic Mutations in Cancer (COSMIC). (A) Following transfection, lysates were probed for CPO expression with a CPO-specific antibody by western blotting. Equal loading was confirmed by Ponceau S staining of the nitrocellulose membrane. (B) Equal amounts of lysate were also incubated with 0.5 mM FA-EE for 30 minutes at 37°C to determine enzymatic activity of each mutant, determined by the decrease in absorbance of the substrate at 340 nm upon cleavage. n = 3, error bars indicate standard error. (TIF) [file pone.0206824.s001.tif]

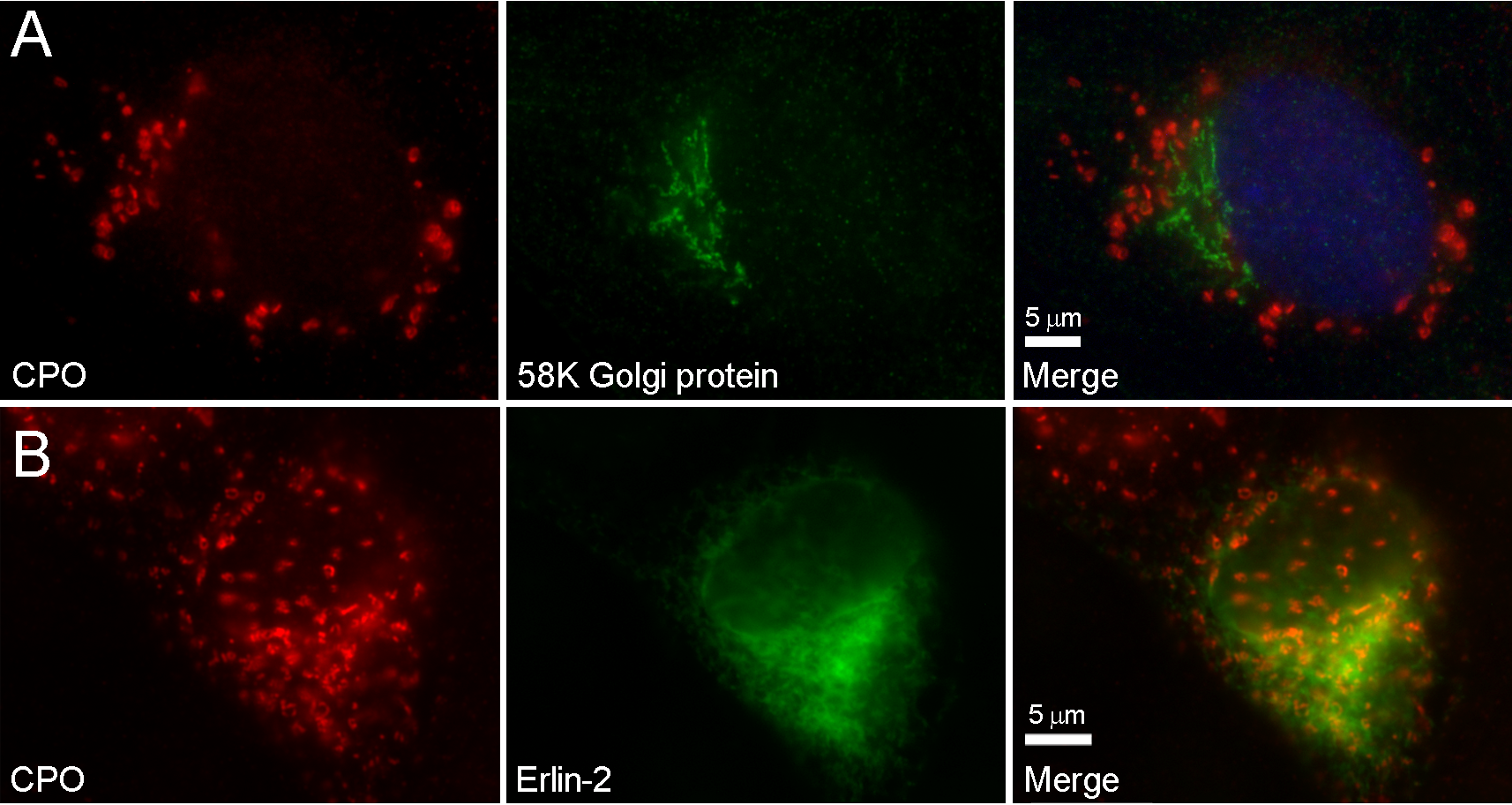

Supplement: S2 Fig — MDCK cells stably expressing CPO were fixed and immunostained with an antibody to CPO (left panels; red) and with 58K Golgi protein (A, green), and erlin-2-GFP (B, green). (TIF) [file pone.0206824.s002.tif]
